# Perceived risk of exposure to indoor residential radon and its relationship to willingness to test among health care providers in Tehran

**DOI:** 10.1186/s40201-014-0118-2

**Published:** 2014-08-23

**Authors:** Narjes Hazar, Mojgan Karbakhsh, Masud Yunesian, Saharnaz Nedjat, Kazem Naddafi

**Affiliations:** Center for Air Pollution Research (CAPR), Institute for Environmental Research (IER), Tehran University of Medical Sciences, Tehran, Iran; Department of Community Medicine, School of Medicine, Tehran University of Medical Sciences, Tehran, Iran; Department of Environmental Health Engineering, School of Public Health, Tehran University of Medical Sciences, Tehran, Iran; Department of Epidemiology and Biostatistics, School of public health, knowledge utilization research center, Tehran University of Medical Science, Tehran, Iran

**Keywords:** Radon, Perceived risk, Risk, Willingness to test, Willingness to pay

## Abstract

**Background:**

Radon exposure is the second cause of lung cancer after exposure to tobacco smoke and the first cause in nonsmokers. The purpose of this study was to assess perceived risk of exposure to indoor residential radon among health care providers in urban and rural health centers affiliated to Tehran University of Medical Sciences.

**Method:**

In 2012–2013, a survey was carried out on 462 health care providers to assess their awareness and risk perception about exposure to indoor residential radon. Only subjects who had previously heard about radon were asked to answer knowledge-based and risk perception questions and report source of knowledge, willingness to test and willingness to pay for radon test kits.

**Results:**

About 67% of responders had heard about radon before this study and of these, 83.5 % recognized it as being hazardous and 34.5 % identified lung cancer as the main health outcome of exposure to radon. Overall, 33% of 310 subjects had knowledgeable awareness. Seventy percent of responders who had previously heard about radon, had high perceived risk and they were more willing to test their houses and more willing to pay for radon test kits.

**Conclusion:**

Having knowledge about radon and perceiving it as a risk had a significant association with willing to take relevant health related behaviors. Furthermore, risk perception contributes to willing to spend more money when health is a concern. Education of health care providers seems to be a pre-requisite to public campaigns on radon awareness and testing.

## Introduction

Exposure to radon is the second cause of lung cancer –just after tobacco smoke- with 3-14% increased risk and the first cause in nonsmokers [1–6]. Radon is a radioactive gas with a half-life of about 3.8 days which arises from soil and rocks and accumulates in mines and houses [4]. It has been shown that exposure to radon has a linear relationship with development of lung cancer [4,7]. Among risk factors ranked according to their attributable burden of diseases, exposure to residential radon comes before diet low in milk, occupational asthmagens and diet high in red meat [8].

In Iran, exposure to residential radon ranks as the 16th risk factor leading to years of life lost (YLLs), and 20th in Disability Adjusted Life Years (DALY). This is while globally, the rank of radon regarding attributable YLLs and DALYs is the 25th [9].

Study of radon awareness and perception has been a public and environmental health issue for at least two decades [10–17]. This category of studies is important as people generally decide to take action against a health hazard according to their understandings of harms and benefits [18]. By definition, risk perception is a subjective assessment about occurrence of an unfavorable event and concerns about the subsequent outcomes [19]. Perceived risk is considered to have three components: personal perception of exposure probability (perceived likelihood), extent of consequent damage (perceived severity) and individual vulnerability (perceived susceptibility) [20]. On the other hand, from the perspective of Health Belief Model [21] six factors are involved in risk perception *i.e*. understanding the existing threats of a condition and promoting individuals to adopt healthy behaviors (model components). These components are “perceived susceptibility”, “perceived severity”, “perceived benefits” (understanding positive outcomes of taking health behavior), “perceived barriers” (as realizing obstacles to health behavior performance), “cues to action” (as reminders of taking health behavior such as TV, other media or an influential informed person) and “self efficacy” (as personal confidence of ability to take positive behaviors or leave the negative ones). Most investigations on a radon awareness and perception have been conducted in developed countries. These studies show that public knowledge and awareness of radon is approximately high in those settings; although, perception and concern about radon health risks has not been as much satisfactory [10–12,22]. In a study from the US, about two third of the general public identified radon’s nature and three quarters of aware individuals recognized it as a health threat [23]. But results of another study there showed that perception of people of radon health hazards was not more than 55% [10]. In contrast, such surveys are scant in developing countries and in the limited published results, researchers have been satisfied with assessment of knowledge and awareness rather than going through perceived risk assessment due to relative unfamiliarity of the public with radon as a health risk [13,14].

Moreover, several developed countries have national plans for monitoring the level of indoor radon and subsequent radon-lowering educations/interventions. For instance, in Finland and Sweden, radon measurement is recommended in months when heating appliances are used more commonly within households. In comparison, in Ireland and Britain, assessment of indoor radon is performed in three months of the year and then the results are adjusted according to the season. As another example, in Italy, yearlong radon measurement is implemented to avoid distortion of results by seasonal variations. In some countries, e.g. the US, measurement of radon is considered as a part of houses’ selling and buying process [4]. It seems that knowledge of Iranians regarding radon as a health risk is scant. In fact, no previous regional or national radon surveys have ever been conducted in Iran to assess the awareness or perceived risk. The limited published reports regarding some local radon assessments in Iran were done with various methods of measurement by engineers and geologists with no implications or associations with health risks or outcomes [24–28]. According to these investigations, about 5-6 percent of houses had elevated levels of radon in some areas, e.g. in north-eastern parts of Iran, if the sample had been representative [27]. We believe that measurement of knowledge and perceived risk of exposure to indoor radon would be the first step of situational analysis in this field. In the first place, we decided to assess public perceived risk of radon. Nevertheless, our first pilot study -that will be discussed further in methods- revealed that public familiarity with radon was too scarce to let us explore their perceived risk. Thus, health care providers in urban and rural health centers affiliated to Tehran University of Medical Sciences were selected for this purpose. We were also interested to examine if this perceived risk was related to their willingness to test and willingness to pay for radon test.

## Methods

A cross sectional study was conducted from July 2012 to February 2013 in Tehran, the capital city of Iran. Participants were all health care providers of entire 54 health care centers providing primary health care (PHC) to the public in the region. These urban and rural health centers are the heath centers of two main health networks in Tehran: “South of Tehran health network” and “Rey health network” (Name of “Rey” or “Ray” comes from the oldest existing city in the province of Tehran). As we found no standard questionnaire to assess radon risk perception in the literature, we developed a self-administered questionnaire based on six components of Health Belief Model to assess perceived risk of radon among participants. This questionnaire consisted of 10 questions in 5-item Likert scale (from completely agree to completely disagree) which would measure “perceived susceptibility”, “perceived severity”, “perceived benefits”, “perceived barriers”, and “self efficacy”. Additionally, three dichotomous questions were designed for assessment of “cues to action” that assessed the source of acquiring the information about radon (media, posters, experts,…). The questionnaire continued with additional questions on willingness to test and willingness to pay for radon test, demographics (age, sex, education) and housing information (house age and duration of residence). We also asked them to rank their concerns about seven health risks: earthquake, radon, and air pollution, exposure to microwave oven, food poisoning, solar radiation and exposure to tobacco smoke. To ensure content validity, the questionnaire was examined by six experts in fields of questionnaire design, environmental health, community health and health education and then necessary modifications were made. The questionnaire was started with the question: “have you ever heard about radon?”. Only participants who answered “yes” to this question were asked to complete the following questions on perceived risk [11,14]. We also included some “knowledge-based questions” about radon e.g. on the nature of radon, being hazardous or not and potential health conditions resulting from radon exposure. "Knowledgeable awareness" was defined as answering correctly to these three questions [13].

Afterwards, two types of available radon test kits (detectors) were explained briefly in the questionnaire (kits for short-term measurement which are kept for about 2 days in place and long-term kits which measure radon level over a season, or even one year). Responders were asked to respond which one they would choose, if they were going to measure radon level in their houses. In addition, they were asked about the maximum amount of money they were willing to pay for indoor residential radon assessment. This can be an important and practical question in our country Iran due to the high percentage of health costs that people have to pay out of the pocket (about 58% in 2001) [29]. In order to assess the maximum amount of money that each person was willing to pay for an indoor radon test, we provided them with four options: up to 500,000(40 US$), 500,000-1000,000(40-80US$), 1000,000-1,500,000 (80–120 US$), 1,500,000 Iranian Rials (120 US$) or more. There was also an option (the fifth) that the responders could choose, if they were willing to test only if it were free. Then, the upper limit of each selected option mentioned above was used as the highest amount of money each subject was willing to pay for indoor residential radon test. In finalizing the questionnaire, the question on “perceived barriers of control” was omitted from the scale as it was found to be confusing with its negative stem and it was not possible to replace it with a positive one without making it more confusing.

Then, in a pilot study, the questionnaire was offered to 50 adults. These people were among general population of Tehran who had come for a visit by health care providers in urban primary health centers. The aims of this pilot study were threefold: firstly, to see if the general public were generally familiar with the issue of radon, so that they could be our potential responders in the main phase of data collection by answering to perceived risk questions. Secondly, to see if the questions were clear enough to be understood by general public and finally, to assess the internal consistency of questions. Among these 50 people, 41 didn't accept to fill out the questionnaire, because of unfamiliarity with radon and lack of interest in the subject. Among remaining, only 3 had heard about radon. At this stage, we were convinced to go one step backwards and choose health care providers of urban and rural health network as our study population, instead. As this latter group were educated in health related fields, we expected that we would have more people with primary knowledge about radon and thus eligible to answer questions on perceive risk. Moreover, health care providers in urban and rural health centers are the first line of preventive health care and education and potentially can become our promoters of radon risk reduction in the community, in the future [30]. Thus, the second pilot study was performed on health care providers in two urban health centers. This time, most of our responders had heard about radon (15 out of 18) and could follow the questionnaire to answer perceived risk questions, as well. We came back 7 days later to conduct retest among our responders. Pearson correlation coefficient of pre- and post- test responses to the main question of the study was 0.76 (P = 0.007). According to post-test results, one of the questions on “perceived susceptibility” was omitted from the scale as deletion was found to improve the Cronbach's alpha from 0.61 to 0.71. Finally we had 8 questions to measure perception of radon risk. Median of scores on perceived risk questions was used in order to calculate “overall median perceived risk” score and the “total score” in SPSS version 16. The associations with demographic characteristics, willingness to test and willingness to pay were assessed using Chi square, Mann–Whitney U and Kruskal–Wallis tests. P value less than 0.05 was considered to be statistically significant. This research was carried out in compliance with the Helsinki Declaration and approved by ethics committee, Deputy of research, Tehran University of Medical Sciences.

## Results

In this study, of 494 individuals eligible to participate, 462 accepted to fill-in the questionnaire (response rate: 93.5%). Demographic and house characteristics of study subjects are demonstrated in Table 1.

**Table 1:** **Demographic and house characteristics of study subjects**

		**Heard about Radon**	**Not heard about Radon**	**P value**
Mean age (SD)	Years	34.47(8.31)	36(8.79)	0.089
Mean years of work (SD)	Years	9.24(7.87)	11.37(8.72)	0.028
Mean house age (SD)	Years	14.23(11.95)	13.05(11.98)	0.225
Mean duration of residence (SD)	Years	6.87(8.17)	5.86(7.51)	0.331
Gender (%)	Male	15.5	11.3	0.216
	Female	84.5	88.7	
	total	100	100	
Education (%)	High school certificate	2.6	10	<0.001
	Associate degree	10.1	22.7	
	Bachelor’s degree	55.8	53.3	
	Masters degree	2.6	0.7	
	Doctoral Degree	28.9	13.3	
	total	100	100	
House status (%)	private	70	65	0.294
	Non private	30	35	
	total	100	100	

About 67% (310 persons) had heard about radon before this study and of these, 88.5% could correctly denote it as a radioactive gas. In addition, 83.5 % of participants recognized it as being hazardous and 34.5 % identified lung cancer as the main health outcome of exposure to radon. Overall, 33% of 310 subjects had “knowledgeable awareness”.

Among seven environmental hazards suggested to rank, the main concern of our study subjects was air pollution (91.5%) followed by earthquake and exposure to tobacco smoke. This is while they ranked the risk of exposure to indoor radon as being of the least importance (38%), even after concern about food poisoning (46.8%) (Table 2). When we selected the subjects who had previously heard about radon, these ranks were the same, except that radon was before the last option (food poisoning) with only 3% difference (48.6 versus 45.6%) (Table 3). For individuals who were knowledgeably aware, concern about radon was as prevalent as 59% compared with 40% for food poisoning; nevertheless, this difference was not statistically significant (P value: 0.268).

**Table 2:** **Concerns of all subjects about seven suggested heath risks**(**n** = **462**)

	**Earthquake**	**Radon**	**Air pollution**	**Food poisoning**	**Solar radiation**	**Microwave**	**exposure to tobacco smoke**
Concerned (%)	75	38	91.5	46.7	59.3	43.4	75
Not concerned (%)	21.5	16	6.5	44.7	32.1	38.8	22
No opinion (%)	3.5	46	2	8.6	8.6	17.8	3
Number (n)	462	431	459	454	454	449	460

**Table 3:** **Concerns of subjects about seven suggested health risks** **in responders who had heard of radon**, **n** = **310**

	**Earthquake**	**Radon**	**Air pollution**	**Food poisoning**	**Solar radiation**	**Microwave**	**exposure to tobacco smoke**
Concerned (%)	76.1	48.6	93.8	45.7	58.6	44	76
Not concerned(%)	19.4	19.5	4.3	45.7	33.9	38.5	21.4
No opinion(%)	4.5	31.9	1.9	8.6	7.5	17.5	2.6
Number (n)	310	298	308	304	307	302	308

Of responders to questions on perceived risk (n = 310), 67.2% considered that it is likely to be exposed to residential radon in Iran, 62.6% believed it is likely that they are exposed to radon in their houses, 54.7% said yes to the question that “It is possible that I develop radon-induced health conditions” and 59% considered the outcome to be serious. Only 9% believed that they could remain healthy, if exposed to radon. About 74% said that radon-induced health-related outcomes can be prevented through radon reducing measures in houses and 50.4% expected that they can reduce radon in their houses with simple and practical actions, if necessary (Table 4). In a spectrum from 1 (meaning no perceived risk) to 5 (high), overall median perceived risk was more than 3 in 70.2% of responders.

**Table 4:** **Perceived risk dimensions**’ **relative frequencies**

	**Completely disagree (%)**	**Disagree to some extent (%)**	**No opinions (%)**	**Agree to some extent (%)**	**Completely agree (%)**
It is likely to be exposed to residential radon in Iran	2	3	27.9	43	24.1
It is possible that I’m exposed to radon in my house	4.3	4.6	28.5	44.2	18.4
It is possible that I develop radon-induced health conditions	3.3	4.6	37.4	40.6	14.1
Residential radon exposure can cause serious diseases in me	3.6	6.6	32.6	36.8	20.4
I am worried about radon to cause serious illness in me	4.5	6	30.5	37.5	21.5
It is possible to prevent radon-induced diseases by reducing its level in houses	2.3	1.3	22.4	36	38
I will remain healthy if I’m exposed to radon due to my good general health status and physical resilience	47	20.5	23.5	6.5	2.5
I can reduce radon in my house with relatively simple and practical actions if necessary	7	6.6	36	30	20.4

Among all responders, 14.1% had their bachelor degrees in occupational and environmental health (9.5% missing). About 93.8% of this subgroup had previously heard about radon. Of these, 40% were knowledgably aware in contrast with 30% in other personnel. In addition, 80% of them had high radon perceived risk. Familiarity with the name of radon was significantly higher among these health providers in comparison to others (P value <0.001) but perception of radon risk was not significantly different (Chi Square P value: 0.065).

We asked participants how they had acquired information about health implications of radon. According to their self-report, in 4.2%, the source of information about health outcomes of radon had been radio/TV, in 8.2% posters, and in 18.4% hearing from an expert (74.4% had benefited from none of the above sources). Long-term radon kits were the most prevalent choice for testing indoor residential radon as 73.5% of subjects who had heard about radon preferred to use it.

Education had a statistically significant relationship with hearing about radon, including all responders in the analysis (P value <0.001). In addition, among subjects who had heard about radon, education was associated with knowledgeable awareness (P value = 0.049): individuals with high school certificate had the lowest level of knowledgeable awareness.

Among responders who had heard about radon, 290 individuals (97%) mentioned that they would accept that indoor radon be measured in their houses. Forty nine percent of them stated that they were willing to test their houses, only if the kits were offered free of charge. In addition, 30% were willing to pay up to 500,000 Rials (40$), and the remaining (21%) were willing to pay even more, if necessary. People with higher perceived risk about radon were significantly more willing to test (P value: 0.022) and more willing to pay for kits (P value: 0.016). In addition, this subgroup seemed ready to pay more money for indoor residential radon test, if necessary (P value: 0.02). Furthermore, women and respondents with knowledgeable awareness were more likely to pay for radon test kits (P value: 0.024 & P value: 0.029, respectively). Subjects who had private houses, had lower level of knowledgeable awareness than those live in rented ones: knowledgeable awareness was 29% in people with private houses in contrast to 43.8% in those living in rented ones (P value:0.013). Age showed no relationship with perceived risk (P value: 0.054) and willingness to pay (P value: 0.986).

Nine responders stated that even if the radon test kits were available free of charge, they wouldn’t use it. Their reasons for non-use were concerns about potential harms of kits, invasion of privacy, not feeling any needs to use it and inability to reduce indoor radon level. Seven out of nine were women and five had bachelor's degree. Six had low perceived risk (overall median perceived risk was 3 or less).

## Discussion

This study was the first to assess indoor residential radon risk perception in Iran. The majority of published papers in this field focus on measuring knowledge and awareness of general public in developed countries [10–12,15,31]. In contrast, our study sample included health care providers working in urban and rural primary health care (PHC) centers in Tehran, Iran. The reason for this choice of study subjects was mainly related to our first pilot study that showed knowledge and awareness about radon was scarce in our general population.

In the current study, similar to the approach of Poortinga *et al*. from the UK [11] and Rahman *et al*. from Pakistan [14], only people who had heard about radon were asked to answer awareness and perceived risk questions, respectively. The justification is that as long as a person has not heard about a risk factor, asking about the awareness and perceived risk might not produce valid results.

Being asked to rank seven environmental risk factors, our responders were most concerned about air pollution. This was not far from expectation; as Iranians especially those residing in Tehran consider air pollution as one of the top priorities among environmental health problems. This project was conducted in Tehran, the capital city of Iran where people are exposed to air pollutants above the permissible levels in several days throughout the year. Thus, the issue is generally among the top health concerns of the society, especially in cold months (months that this study was conducted) [32,33]. Moreover, It has been demonstrated that when a hazard is more presented and discussed through media, it will be more available to the minds of people as a real threat in comparison with the setting that the same risk factor is a neglected and marginal issue [34]. In our study, one of the other most feared hazards was earthquake. As demonstrated in previous reports [34], people are more afraid of catastrophic events such as earthquake -that kill many in a few seconds- than chronic incidents such as cancers. In addition, Tehran is located in a seismic zone and unfortunately, several earthquakes have been recorded for this Metropolitan city [35].

In individuals who had heard about radon before the study, concern about radon obtained the sixth rank with only 3% difference with food poisoning (as the 7th). This is while, according to the latest global burden of disease report, attributable years of life lost (YLL) and Disability Adjusted Life Years (DALY) of exposure to residential radon are higher than those of exposure to ozone, household air pollution or unimproved water [9]. These findings show that our study population does not consider indoor radon exposure to be among their first priorities in the context of environmental hazards.

More than half of subjects had heard about radon and of them, 88.5% recognized it as a radioactive gas. In a study from Pakistan, 20.2% of general population knew radon and 17.4% considered it as a health threat [13]. Another study from Pakistan showed that none of uneducated individuals were familiar with radon and this was about 30% in educated people [14]. This is while, in studies from the UK and the US, the knowledge levels were much higher (72 & 96%, respectively) [10,11]. In addition, while one third of our responders declared that radon may cause lung cancer, more than 50% of rural residents in US were aware about this important outcome [10,23]. Moreover this finding was similar to the report by Rafique *et al*. from Pakistan [13].

These comparisons suggest that in developing countries, not only the general populations are not familiar with the risks of exposure to indoor radon, but also awareness of health personnel is scant and remarkably lower than those in developed countries.

In this project like two other reports by Wang and Brown [15,36], individuals who were more educated, had more awareness about radon. Knowledgeable awareness didn’t follow such a trend and was highest in bachelors than medical doctors. This may be due to the fact that some of our responders with BS/BA degrees in health sciences had their bachelors in environmental and occupational health. Moreover, environmental health issues might be more emphasized in curricula of allied heath majors than in medicine.

It seems that indoor radon is not recognized as an environmental risk among the public, health care providers and researchers in Iran. Nearly a half of heath care workers familiaret with radon were concerned about it. In addition, when we asked them about their willingness to pay for testing their houses, nearly half of them emphasized that they would use radon test kits only if they would be provided free of charges. These results are in line with some previous reports [10–12,15] showing that high awareness of health risks doesn’t essentially result in high concern in population.

Among our participants, more than two thirds had high perceived risk. In the study by Duckworth and colleagues, conducted on a sample of general population in the US, this was about 55% [10]. More responders with high perceived risk in our study in comparison with Duckworth's research might be due to the difference of responders regarding education and work field.

When we asked responders about the source of radon information, only a small number of aware participants had received this information through the radio/TV whereas 85% of American general population said that they got informed through the news [15]. Media can have an important role to transfer health related information to the public [37]. Scientific programs in media have significantly increased in recent years in Iran [38] but; unfortunately some issues like radon are still neglected [39].

As radon test kits are not still readily available to the public in Iran, willingness to test was assessed only by asking one question. Among participants, the higher the perceived risks, the higher the willingness to test their houses for indoor radon. There is a significant association between each dimension of risk perception (perceived likelihood, perceived susceptibility and perceived severity) and taking health behaviors [20]. Thus, when general awareness and risk perception is low, people might not be interested to take part in such surveys. Thus, if public health sector intends to run nationwide surveys of indoor radon assessment, campaigns for improving relevant knowledge and perceived risk is a pre-requisite. We can expect that if people are more informed about potential risks of exposure to indoor radon, they would be more willing to test their houses for radon level.

Most of the responders who were willing to test their houses for radon, preferred long term test kits (3–6 month), if provided. These detectors which are the most accurate among options for radon detection [4] are used in some developed countries such as Canada for national surveys [40,41]. On the other hand, they need to be kept in place for a considerably long period of time [4]. Since our study participants showed interest in long term radon test kits which have been used in national surveys in developed countries, it is expected that these types of kits can be employed successfully in future surveys in Iran.

Most of our aware respondents were willing to test their houses, only if the kits were offered free of charge. In addition, willing to pay showed a significant relationship with knowledgeable awareness and perceived risk. A survey in Britain demonstrated that most of responders who had high perceived risks of climate change were willing to pay more money to use renewable energy sources in order to avoid subsequent disadvantages [42]. Another survey among UK and US residents demonstrated that risk perception of genetically modified foods has a positive association with willing to pay more money to use natural foods without genetic modification [43]. It seems that people are willing to pay for a service or product, if they have positive previous experience about its benefits and/or know non-use of it might be a risk for health [44,45]. Women were more likely to say that they are willing to pay for kits. This result is consistent with another study that women were more interested to spend money for radon mitigation in their houses [46]. This might show that there is a higher tendency in women to invest for healthy conditions [47], even when they are contributors to the family income (e.g. health care providers).

In our setting, people living in rented houses had more knowledgeable awareness than those who had private ones. This is a finding that cannot be easily explained, as others eg Larsson *et al*. observed that house owners were more aware than other groups [31]. One of the reasons for our finding might be the complexity of association between socio-economic status and education in Iran [48].

One of the strong points of the current research is that as the first study on this environmental risk factor in Iran, various aspects of perceived risk were assessed according to Health Belief Model (HBM). In this study we couldn’t assess general public risk perception of radon because of the low knowledge and awareness observed in our first pilot study. Community education about such silent killers is necessary to avoid excess attributed mortality.

## Conclusion

Radon as the second most important risk factor of lung cancer was a neglected issue even among health care providers. They did not consider indoor radon exposure to be among their first priorities in the context of environmental hazards. Moreover, one third of them declared that radon may cause lung cancer. Nearly half of heath care providers familiar with radon were concerned about it. In addition, when we asked them about their willingness to pay for testing their houses for radon, nearly half of them emphasized that they would use radon test kits only if they would be provided free of charges. Willingness to pay showed a significant relationship with knowledgeable awareness and perceived risk.

Having knowledge about health hazards like radon and perceiving them as a risk has a positive relationship with taking health related behaviors. Furthermore, risk perception contributes to spending more money when health is a concern.
